# ED Patient Navigators: Enhancing emergency care for older adults & dementia patients

**DOI:** 10.1002/alz70858_098555

**Published:** 2025-12-25

**Authors:** Eric D Isaacs

**Affiliations:** ^1^ Zuckerberg San Francisco General Hospital, San Francisco, CA, USA

## Abstract

This session will describe how the Zuckerberg San Francisco General Hospital and Trauma Center (ZSFG), an urban, county public hospital and trauma center, integrated and implemented geriatric and dementia care practices into emergency care for older adults and persons living with dementia. There will be discussion about how non‐healthcare workers are trained, established, and integrated into the existing emergency department workflow to function as Patient Navigators providing person‐centered emergency care by screening and identifying older adults and those with dementia to ensure what matters most in patient care is achieved.

The development of Geriatric Emergency Departments (GED) centers around use of nurses or advance practice providers to screen for geriatric syndromes and connect patients to community resources and follow‐up care. In our public hospital, we began implementation of a GED program with an eye on sustainability marked by highly effective interventions with less budgetary impact. The novel use of a lay person Geriatric Patient Navigator is such an intervention.

The GED patient navigator (PN) is a post‐baccalaureate candidate interested in the health professions. Patients are screened for frailty using the ISAR and highlighted on the tracking board for the PN to see if ISAR positive. The PN was trained in screening tools for common geriatric syndromes, including cognitive impairment, mobility, ADLs, depression, nutrition, and caregiver stress. Appropriate resources addressing these geriatric syndromes were compiled and selected for patient referral and follow‐up at discharge.

Patients are screened by nurses for fall risk and risk of suicide by nurses at triage, and screened for delirium by the bedside nurse. Comprehensive depression screen and caregiver stress screens are performed by a dedicated social worker. Utilization management nurses are available to match discharge resources with organizations and providers accepting that particular patient's insurance. The Patient Navigator performs phone follow‐up with discharged patients in 24‐72 hours to ensure provided resources were accessed and to elicit and difficulties encountered since discharge.

The majority of patient navigator tasks do not require licensed personnel. Using a patient navigator to perform the abundance of non‐clinical tasks and outside communication will preserve nursing and provider resources for direct patient care.

